# The value of CDX2 and cytokeratins 7 and 20 expression in differentiating colorectal adenocarcinomas from extraintestinal gastrointestinal adenocarcinomas: cytokeratin 7-/20+ phenotype is more specific than CDX2 antibody

**DOI:** 10.1186/1746-1596-7-9

**Published:** 2012-01-23

**Authors:** Reyhan Bayrak, Hacer Haltas, Sibel Yenidunya

**Affiliations:** 1 Department of Pathology, Fatih University Hospital, Ankara, Turkey

**Keywords:** gastrointestinal adenocarcinomas, CK7, CK20, CDX2, immunohistochemistry

## Abstract

**Background/Objective:**

Metastatic adenocarcinoma from an unknown primary site is a common clinical problem. Determining the cytokeratin (CK) 7/CK20 pattern of tumors is one of the most helpful procedures for this purpose since the CK7-/CK20+ pattern is typical of colorectal adenocarcinomas. CDX2, a critical nuclear transcription factor for intestinal development, is expressed in intestinal epithelium and adenocarcinomas. In the present study, we compared the sensitivity and specificity of CDX2 expression and the CK7-/CK20+ phenotype in differentiating colorectal adenocarcinomas from pancreatic and gastric adenocarcinomas.

**Methods:**

CK7/CK20 staining pattern and CDX2 expression were evaluated in 118 cases of colorectal, 59 cases of gastric, and 32 cases of pancreatic adenocarcinomas. The sensitivity, specificity, and positive and negative predictive values of the CK7-/CK20+ phenotype and of CDX2 expression were analyzed.

**Results:**

The CK7-/CK20+ immunophenotype was expressed by 75 of 118 (64%) colorectal and 3 of 59 (5%) gastric tumors and was not observed in any pancreatic adenocarcinomas. The CK7+/CK20+ immunophenotype was expressed in 24/118 (20%) of colon, 28/59 (48%) of gastric and 7/32 (22%) of pancreatic adenocarcinomas. The CK7+/CK20- expression pattern was observed in only 2% (2 of 118) of colorectal carcinomas. CDX2 was expressed in 114 of 118 (97%) colorectal, 36 of 59 (61%) gastric, and 5 of 32(16%) pancreatic adenocarcinomas. There was no significant association between CDX2 expression and tumor differentiation in colorectal carcinomas. In gastric carcinomas, CDX2 expression was more common in intestinal type tumors than in diffuse type carcinomas. The CK7-/CK20+ phenotype showed a specificity of 96.7% in predicting colorectal adenocarcinomas, which was superior to that of CDX2 expression. CDX2 expression at both cut-off levels (> 5% and > 50%) had a higher sensitivity (96.6% and 78%) than the CK phenotype.

**Conclusions:**

Both the CK7-/CK20+ phenotype and expression of the antibody CDX2 are highly specific and sensitive markers of colorectal origin. CDX2 expression should be a useful adjunct for the diagnosis of intestinal adenocarcinomas, particularly when better established markers such as CK7 and CK20 yield equivocal results. The CK7-/CK20+ phenotype is superior in its specificity and positive predictive value and might be preferred.

**Virtual slides:**

The virtual slide(s) for this article can be found here:

http://www.diagnosticpathology.diagnomx.eu/vs/4851011866354821

## Background

Metastatic adenocarcinoma from an unknown primary site is a common clinical problem that leads to extensive and costly clinical and radiological examinations, sometimes with discouraging results [1,2]. It is often important to determine the site of origin of a metastatic carcinoma of unknown primary site, particularly because this may affect the choice of the treatment. A more precise diagnosis leads to more effective treatment, substantially improving the overall outcome [3]. Determination of the primary site may take several steps. Clinical features, such as age, sex, and site of metastases may give a first indication. The histological assessment is often very helpful, but may not differentiate adequately between various primary tumors. Immunohistochemistry is the most common adjunctive method used in the analysis of the patient with cancer of unknown primary site [4,5].

Cytokeratins (CKs) represent the epithelial class of intermediate-sized filaments of the cytoskeleton. There are 20 subtypes of cytokeratin (CK) intermediate filaments. These have different molecular weights and demonstrate differential expression in various cell types and tumors [6]. Among the most useful cytokeratins are CK7 and CK20 [7]. CK7 is found in many ductal and glandular epithelia, including lung, breast, ovary, and endometrium [8,9]. CK20 is expressed in the gastrointestinal (GI) epithelium, urothelium, and Merkel cells [10]. The combined expression patterns of CK7 and CK20 have been extensively studied in various primary and metastatic carcinomas [5,7,11-17]. CK20 is expressed alone in the majority of intestinal adenocarcinoma and in Merkel cell carcinomas whereas CK7 is present without CK20 in most breast, lung and ovarian adenocarcinoma, and with CK20 in urothelial, pancreatic and gastric carcinomas. The CK7-/CK20+ expression pattern is known to be highly characteristic of colorectal carcinomas [11,12,17-19], however, not all colorectal carcinomas show the CK7-/CK20+ expression pattern. Occasionally colorectal carcinomas may show significant CK7 expression and conversely, expression of CK20 may be seen in a variety of non-colorectal adenocarcinomas such as urothelial, gastric and pancreatobiliary tract carcinomas [20-24]. For this reason, there is continued interest in the development of new and more specific markers of intestinal differentiation and CDX2 appears to be such a marker.

CDX2 is a caudal-type homeobox gene, encoding a transcription factor that plays an important role in proliferation and differentiation of intestinal epithelial cells [25]. The protein (CDX2) is normally expressed throughout embryonic and postnatal life within nuclei of intestinal epithelial cells from the proximal duodenum to the distal rectum [26,27]. Previous studies showed that CDX2 is expressed in normal and neoplastic intestinal epithelial cells with a relatively high sensitivity and specificity and that it can be used as an immunohistochemical marker for neoplasms of intestinal origin [28-32]. However, CDX2 expression was also found in gastric carcinoma, and other carcinomas with intestinal-type morphology [33-36].

In the present study, we examined the expression profiles of CK7, CK20, and CDX2 immunohistochemical markers in primary colorectal, gastric and pancreatic adenocarcinomas in consideration of the potential applicability of these markers in the clinical context of metastatic adenocarcinomas. We also evaluated the sensitivity, specificity, positive predictive value, and negative predictive value of CDX2 expression and CK7-/CK20+ immunophenotype to differentiate colorectal adenocarcinomas from pancreatic and gastric adenocarcinomas.

## Materials and methods

### Case selection and tissue samples

One hundred eighteen colorectal, 59 gastric and 32 pancreatic adenocarcinoma resection specimens were retrieved from the archival files of the Department of Pathology, Fatih University Medical School, between January 2006 and December 2009. Pathological findings, including histological type, histological differentiation, depth of invasion, and lymph node status, were gathered from hematoxylin and eosin stained sections. All cases were reviewed to confirm the diagnosis. The grade and histological type of colorectal and pancreatic adenocarcinomas were determined according to criteria of the World Health Organization (WHO) Classification of Tumors [37]. Well and moderately differentiated tumors were grouped together as low-grade tumors and were compared with high-grade tumors, which included poorly differentiated and undifferentiated tumors, and signet ring cell carcinomas. Histological typing of gastric carcinomas was made according to Lauren classification [38]. Adenocarcinomas of intestinal type, which were well or moderately differentiated, were recorded as low grade tumors, whereas the poorly differentiated intestinal type adenocarcinomas and the diffuse type adenocarcinomas were recorded as high grade tumors. Postoperative pathological staging was performed according to the American Joint Committee on Cancer (AJCC) TNM staging system [39]. One paraffin block with the maximum amount of tumor and proper fixation was selected from each case for immunohistochemical studies. This study was approved by Ethics Committee of Fatih University Hospital (09.23.2010/B 302 FTH 0200000)

### Immunohistochemical Analysis

Four μm-thick sections were cut from blocks of paraffin embedded tissue, deparaffinized, and rehydrated as usual. To reduce non-specific background staining due to endogenous peroxidase, slides were incubated in Hydrogen Peroxide Block for 15 min. Before immunostaining, antigen retrieval was performed by incubating the slides for 15 min with pepsin (LabVision; catalog no. AP-9007) at a concentration of 1mg/ml for CK20. Slides

were microwaved in 10 mM of citric acid at pH6.0 for 20 min for CK7 and CDX2. The slides were incubated for 60 min with primary antibodies to CK7 (clone OV-TL 12/30, LabVision/NeoMarkers; 1:50), CK20 (clone Ks20.8, Dako; 1:50) and CDX2 (clone AMT 28, NovoCastra; 1:50) at room temperature. The Standard avidin-biotin-peroxidase complex (ABC) technique was performed using the LabVision Secondary Detection Kit (UltraVision Detection System Anti-polyvalent, HRP). AEC was used as chromogen. All slides were counter stained with Mayer's hematoxylin.

### Microscopic Evaluation

For CDX2, only nuclear staining was considered positive. Cytoplasmic positivity was infrequently encountered, and was considered an artifact. Positive immunostaining for CK7 and CK20 was identified in the cytoplasm, cell membrane, or both. The percentage of positive cells was scored in a semiquantitative method according to the following scheme: 0 (less than 5% of tumor cells); 1+ (positive signal of any intensity in 5-25% of tumor cells); 2+ (26-50% of tumor cells); 3+ (51-75% of tumor cells); and 4+ (greater than 75% of tumor cells). Furthermore, staining in less than 50% of the tumor cells was considered focal, and staining in more than 50% of the tumor cells was considered diffuse positivity. In general, cases showing 3+ and 4+ staining also had strong intense staining, so intensity was not used in determination of the final reactivity score. Normal colonic mucosal tissue was used as a CK20 and CDX2-positive control, and normal pancreatic tissue was used as a CK7-positive control. For negative control samples, the primary antibody was omitted for each run.

### Statistical analysis

χ^2^and Fisher exact tests were used to compare the differences in percentages of positive results between groups. SPSS 13.0 for Windows was used for all statistical analyses. The sensitivity, specificity, and positive and negative predictive values of the CK7-/CK20+ phenotype and of CDX2 expression were counted.

## Results

Tables 1 and 2 show the percentage of cases that stained with CDX2, CK7, and CK20 in colorectal adenocarcinomas, gastric adenocarcinomas and pancreatic adenocarcinomas.

**Table 1:** Distribution of CK7, CK20 and CDX2 staining with percentages of positive cells in primary colorectal, gastric and pancreatic adenocarcinomas

		Negative	Positive				Total
				
		0	1+	2+	3+	4+	
**Colorectal adenocarcinoma**	**CK7**	92 (78%)	8 (7%)	6 (5%)	5 (4%)	7 (6%)	118
	**CK20**	19 (16%)	20 (17%)	16 (13%)	22 (19%)	41 (35%)	118
	**CDX2**	4 (3%)	7 (6%)	15 (13%)	21 (18%)	71 (60%)	118
**Gastric adenocarcinoma**	**CK7**	12 (20%)	5 (9%)	7 (12%)	6 (10%)	29 (49%)	59
	**CK20**	28 (47%)	16 (27%)	6 (10%)	8 (14%)	1 (2%)	59
	**CDX2**	23 (39%)	13 (22%)	9 (15%)	10 (17%)	4 (7%)	59
**Pancreatic adenocarcinoma**	**CK7**	1 (3%)	1 (3%)	1 (3%)	9 (28%)	20 (63%)	32
	**CK20**	25 (78%)	6 (20%)	1 (3%)	0 (0%)	0 (0%)	32
	**CDX2**	27 (85%)	3 (9%)	2 (6%)	0 (0%)	0 (0%)	32

**Table 2:** CK7/20 phenotype and CDX2 expression in our studied groups

	Colorectal AdenoCa (n = 118)		Gastric AdenoCa (n = 59)		Pancreatic AdenoCa (n = 32)	
			
	CDX2+	CDX2-	CDX2+	CDX2-	CDX2+	CDX2-
**CK7-/CK20+**	74 (62%)	1 (1%)	3 (5%)	0 (0%)	0 (0%)	0 (0%)
**CK7+/CK20+**	22 (19%)	2 (2%)	21 (36%)	7 (12%)	2 (6%)	5 (16%)
**CK7+/CK20-**	2 (2%)	0 (0%)	8 (14%)	11 (19%)	3 (9%)	21 (66%)
**CK7-/CK20-**	16 (14%)	1 (1%)	4 (7%)	5 (9%)	0 (0%)	1 (3%)

### CK7 and CK20

**Figure 1:**
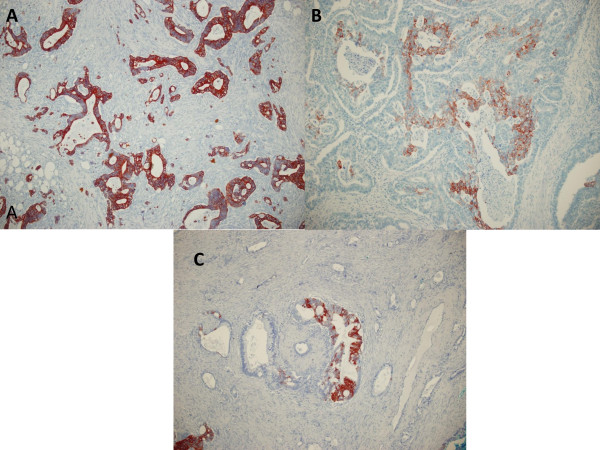
**CK20 staining in colorectal, gastric, and pancreatic adenocarcinomas**. (A) Colorectal adenocarcinoma displayed diffuse and strong immunoreactivity in the cytoplasm of cancer cells. (B) Gastric and (C) pancreatic adenocarcinomas exhibited focal cytoplasmic staining for CK20. Original magnification × 100.

**Figure 2:**
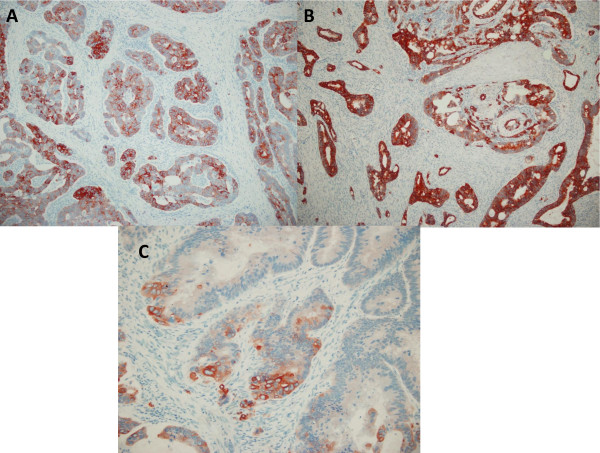
**CK7 immunostaining in colonic, gastric, and pancreatic tumors**. (A) Gastric and (B) pancreatic carcinomas showed diffuse and strong positive CK7 immunostaining. (C) Colonic adenocarcinoma displayed focal cytoplasmic immunoreactivity for CK7. (A)-(B), original magnification × 100; (C), original magnification × 200.

CK7 expression was detected in 22% (26/118) of colorectal, in 80% (47/59) of gastric, and in 97% (31/32) of pancreatic adenocarcinomas. CK20 reactivity was found in 84% (99/118) of colorectal, in 53% (31/59) of gastric, and in 22% (7/32) of pancreatic adenocarcinomas. The CK7-/CK20+ immunophenotype was expressed by 75 of 118 (64%) colorectal and 3 of 59 (5%) gastric tumors and was not observed in any pancreatic adenocarcinomas (χ^2 ^= 79.992; p < 0.001). The CK7+/CK20+ immunophenotype was expressed in 24/118 (20%) of colon, 28/59 (48%) of gastric and 7/32 (22%) of pancreatic adenocarcinomas, which was not helpful in the differential diagnosis. However, among the CK20 positive cases, CK20 reactivity was diffuse (more than 50% of cells were positive) in the majority of colorectal carcinomas in 64% (63/99) of the cases and mainly focal ( < 50% of cells were positive) in gastric and pancreatic adenocarcinomas in 71% (22/31) and 100% (7/7) of cases respectively (χ^2 ^= 19.509; p < 0.001) (Figure 1). Conversely, among the CK7 positive cases, CK7 reactivity was diffuse in the majority of gastric and pancreatic adenocarcinomas in 74% (35/47) and 94% (29/31) of cases respectively, and this reactivity was focal in 54% (14/26) of colorectal carcinomas (χ^2 ^= 16.228;p < 0.001) (Figure 2). The CK7+/CK20- expression pattern was observed in only 2% (2 of118) of colorectal carcinomas, although it was expressed in 32% (19/59) of gastric and 75% (24/32) of pancreatic adenocarcinomas (χ^2 ^= 85.607; p < 0.001). In our study, 17(14%) colorectal, 9 (15%) gastric, and only 1 (3%) pancreatic adenocarcinomas showed a CK7-/CK20- immunophenotype.

CK7 and CK20 expression were compared with the clinicopathological characteristics of the tumors (Table 3). No association between CK7 expression and anatomical location of carcinomas, tumor type, stage, and grade was found. No association was observed among CK20 expression and tumor type, tumor stage (pT), or nodal status. Among the colorectal tumors, CK20 positivity was more common in rectal carcinomas than in nonrectal colon carcinomas (89% versus 70%, χ^2 ^= 6.839; p = 0.009) and in low grade carcinomas than in high grade carcinomas (91% versus 55%, χ^2 ^= 17,247; p < 0.001).

**Table 3:** Expression of CK7, CK20, and CDX2 in cancer tissues by histopathological characteristics

	CK7+		CK20+		CDX2+	
			
	n	%	n	%	n	%
**Colorectal adenocarcinoma (n = 118)**						
Low grade (n = 96)	22	22.9	87	90.6 *	94	97.9
High grade (n = 22)	4	18.2	12	54.5 *	20	90.9
Rectal (n = 85)	18	21.2	76	89.4§	83	97.6
Nonrectal (n = 33)	8	24.2	23	69.7§	31	93.9
**Gastric adenocarcinoma (n = 59)**						
Intestinal type (n = 30)	21	70.0	16	53.3	23	76.7 #
Diffuse type (n = 29)	26	89.7	15	51.7	13	44.8 #
**Pancreatic adenocarcinoma (n = 32)**						
Low grade (n = 30)	30	100.0	7	23.3	5	16.7
High grade (n = 2)	1	50.0	0	0.0	0	0.0

* p < 0.001 § p = 0.009 # p = 0.012

### CDX2

**Figure 3:**
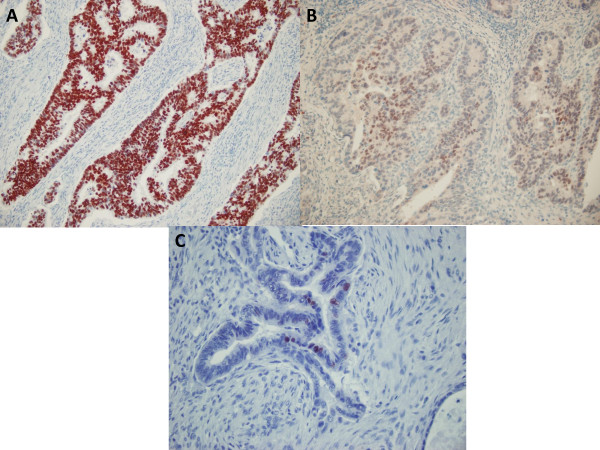
**CDX2 expression patterns in colorectal, gastric, and pancreatic carcinomas**. (A) Homogeneous (diffuse and strong) nuclear expression of CDX2 in a low grade colorectal adenocarcinoma. (B) Gastric and (C) pancreatic adenocarcinomas showed heterogeneous pattern of intensity of CDX2 expression, frequently encountered in extra-intestinal GI adenocarcinomas. (A)-(B), original magnification × 200; (C), original magnification × 400.

CDX2 was expressed in 114 of 118 (97%) colorectal, 36 of 59 (61%) gastric, and 5 of 32 (16%) pancreatic adenocarcinomas (χ^2 ^= 93.576; p < 0.001). In positive cases, the immunoreactivity was predominantly nuclear with occasional faint cytoplasmic staining. The majority of cases (92/114, 81%) demonstrated strong and diffuse immunostaining in more than 50% of cells in colorectal tumors. Among the CDX2 positive gastric carcinomas (36/59), reactivity was focal in 22 cases (22/36, 61%). Among the 32 cases of pancreatic adenocarcinoma, only 5 cases were focally positive for CDX2 (χ^2 ^= 33.462; p < 0.001) (Figure 3).

**Figure 4:**
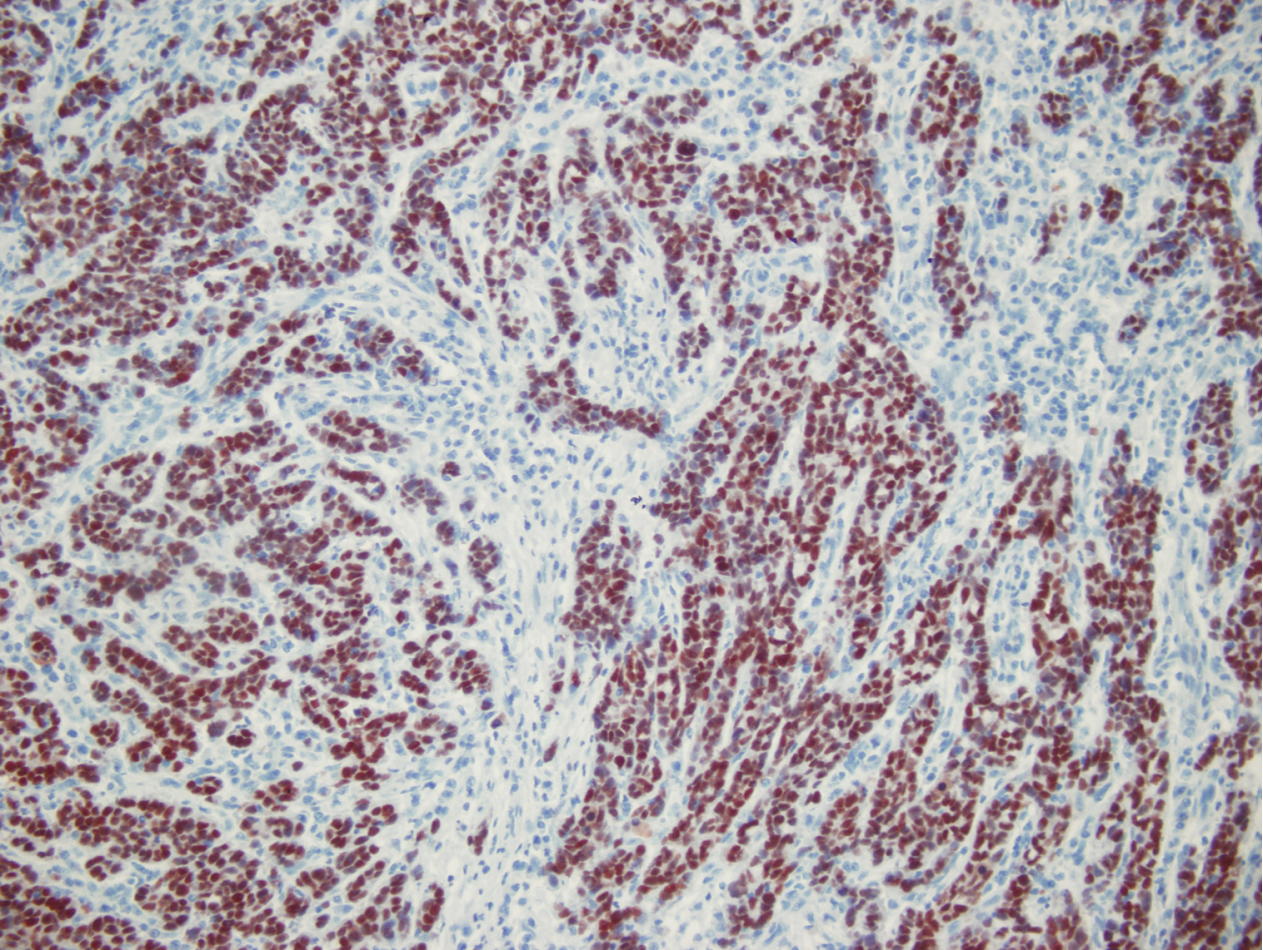
**CDX2 expression in a high grade colorectal adenocarcinoma**. Diffuse moderate to strong nuclear staining was seen. Original magnification × 200.

CDX2 expression was also compared with the clinicopathological characteristics of the tumors (Table 3). In gastric carcinomas CDX2 expression was more common in intestinal type tumors than in diffuse type carcinomas (77% versus 45%, χ^2 ^= 6.284; p = 0.012). There was no significant association between CDX2 expression and tumor differentiation in colorectal carcinomas (98% of low grade tumors and 91% of high grade tumors were positive for CDX2) (Figure 4). Conversely, among gastric carcinomas CDX2 positivity was more common in low grade carcinomas than in high grade carcinomas (80% versus 51%, χ^2 ^= 4.584; p = 0.032). No association was observed among CDX2 expression and anatomical location of carcinomas, tumor stage (pT), or nodal status.

### Comparison of CK7/20 staining pattern and CDX2 expression

The CK7-/CK20+/CDX2+ phenotype was highest, accounting for 63% (74/118) of colorectal adenocarcinomas. In gastric and pancreatic adenocarcinomas, CK7+/CK20+/CDX2+ (21/59, 36%) and CK7+/CK20-/CDX2- (21/32, 66%) were the most common patterns respectively. In CK7+/CK20+ tumors, CDX2 expression was observed in 22 of 24 (92%) colorectal, 21 of 28 (75%) gastric, and 2 of 7 (29%) pancreatic carcinomas. This reactivity was diffuse in majority of colorectal carcinomas in 68% (15/22) of the cases and mainly focal in gastric and pancreatic adenocarcinomas in 57% (12/21) and 100% (2/2) of cases respectively (χ^2 ^= 5.979; p = 0.051). Among the CK7-/CK20- colorectal tumors CDX2 was positive in 16 of 17 (94%) cases.

We also evaluated the sensitivity, specificity, positive predictive value, and negative predictive value of CDX2 expression and CK7-/CK20+ immunophenotype to differentiate colorectal adenocarcinoma from pancreatic and gastric adenocarcinomas (Table 4). Determining the CK7/CK20 phenotype proved to be more specific in differentiating colorectal adenocarcinoma from pancreatic and gastric adenocarcinomas (specificity 96.7%) than the expression of CDX2 was. The CK7-/CK20+ phenotype had a superior positive predictive value (96.2%) in these circumstances. CDX2 expression at both cut-off levels (> 5% and > 50%) had a higher sensitivity (96.6% and 78%) and higher negative predictive value (92.6% and 74.8%) than the CK phenotype. The specificity of CDX2 expression did not reach the level of specificity of CK7/CK20 phenotype at a > 50% level, either (84.6%).

**Table 4:** Sensitivity, specificity, and positive/negative predictive values of CDX2 expression and CK7-/CK20+ immunophenotype in differentiating colorectal adenocarcinomas from pancreatic and gastric adenocarcinomas.

	Sensitivity	Specificity	PPV	NPV
**CK7-/CK20+**	63.6%	96.7%	96.2%	67.2%
**CDX2 > 5%**	96.6%	54.9%	73.5%	92.6%
**CDX2 > 50%**	78.0%	84.6%	86.8%	74.8%

**PPV**: Positive Predictive Value **NPV**: Negative Predictive Value

## Discussion

The diagnosis of the metastatic carcinoma of unknown origin can be very difficult. The determination of the primary site of the metastasis is a challenge to both oncologists and pathologists, having potentially important clinical and therapeutic consequences [1-3]. In the setting of carcinomas of unknown primary, clinicopathological correlation and a panel of standard immunostains help define the primary site, and direct appropriate treatment [4,5].

Cytokeratins are group of approximately 20 proteins that consist of a type of intermediate filament and are differentially expressed in epithelia of various sites. The cytokeratins most often used are CK7 and CK20 [7-10]. CK7 is found in the glandular epithelium and epithelial tumors of lung, ovary, endometrium and breast, but is not found in GI epithelium. Conversely, CK20 is expressed principally in the normal glands and epithelial tumors of the GI tract, urothelium, and Merkel cells. The cytokeratin 7/20 profile of a particular tumor has proved to be a useful aid in differential diagnosis of carcinomas, since primary and metastatic tumors tend to retain the cytokeratin profiles of the epithelium from which they arise [13]. In his review article, Tot summarized the results of 29 studies containing more than 3500 reported cases of adenocarcinomas stained with CK20 and CK7. This review stated that metastatic colorectal, gastric and pancreatic adenocarcinomas have similar CK7 and CK20 staining ratios as their respective primary tumors. Only gastric adenocarcinomas showed statistically significant differences in CK20 expression when the primary and metastatic locations were compared [13].

Normal epithelium of the small bowel, appendix and colorectum, and adenocarcinomas from these sites, are almost consistently CK7-/CK20+, helping to distinguish these adenocarcinomas from adenocarcinomas of many other primary sites [9-15]. The CK7-/CK20+ pattern was identified in 65% to 95% of the colorectal adenocarcinomas in different series [11,12,20-23,31]. On the other hand approximately one third of gastric and less than 10% of pancreatic adenocarcinomas also show this pattern [11,12,23]. The CK7-/CK20+ immunophenotype was expressed by 75 of 118 (64%) colorectal and 3 of 59 (5%) gastric tumors and was not observed in any pancreatic adenocarcinomas in the present study. Therefore, it has been presumed that CK7 is not typically expressed by colonic epithelial tumors. Interestingly, several reports have described CK7 expression in colorectal adenocarcinoma, with expression ranging from 5% to 74% [11,12,22,23,31]. The reasons for this discrepancy are unclear. However, this may be the result of differences in the studied population or the interpretive criteria that was used. In our study, CK7 expression was detected in 22% (26/118) of colorectal adenocarcinomas.

In comparison with the CK7-/CK20+ immunoprofile, the CK7+/CK20+ immunoprofile is commonly present in urothelial carcinomas, gastric carcinomas and tumors of the pancreatobiliary tract [11,12,15]. Gastric adenocarcinomas are the most heterogeneous subgroup of carcinomas with respect to their CK7/CK20 immunophenotype. While most gastric adenocarcinomas are CK20+, they may or may not be CK7+ [11,12,23]. The results of CK7/CK20 immunohistochemistry for cholangiocarcinomas, gall bladder carcinoma and pancreatic ductal adenocarcinoma are conflicting. While all studies have found CK7 immunopositivity in these tumours, many studies have found the majority are CK20- [40,41], while others have found the majority to be CK20+ [11,12]. In the present study the largest proportion of gastric carcinomas was of the CK7+/CK20+ phenotype (48%), and a substantial proportion was of the CK7+/CK20- phenotype (32%). CK7+/CK20- immunoprofile was the most common pattern, accounting for 75% of pancreatic adenocarcinomas. The CK7+/CK20+ immunophenotype was expressed in 20% of colon, 48% of gastric and 22% of pancreatic adenocarcinomas, which was not helpful in the differential diagnosis. However, CK20 reactivity was diffuse (more than 50% of cells were positive) in the majority of colorectal carcinoma cases and mainly focal ( < 50% ofcells were positive) in gastric and pancreatic adenocarcinomas as in previous studies [22,23,31,41].

Since, occasional colorectal carcinomas may show significant CK7 expression and conversely, expression of CK20 may be seen in a variety of non-colorectal adenocarcinomas, there is interest in the development of new and more specific markers of intestinal differentiation. Human CDX2 protein is a member of the homeobox genes that encodes an intestine-specific transcription factor. This protein regulates intestinal development and is expressed in the nuclei of epithelial cells throughout the intestinal tract in embryonic and postnatal life [25-27]. Expression of CDX2 mRNA has been shown to be highly restricted to intestinal epithelium [42]. The sensitivity and specificity of antibodies to CDX2 protein as a marker of colonic adenocarcinoma has been recently evaluated in various studies with reported sensitivity and specificity of greater than 90% [28-33]. Werling et al [28] examined CDX2 expression across 476 samples of human tumors and concluded that it is an excellent marker of adenocarcinomas arising in the GI tract, particularly the duodenum and colon. These authors reported that high levels (> 75% positive cells) of CDX2 expression were found almost exclusively in adenocarcinomas of the colorectum, and intermediate levels (26%-75% positive cells) of immunostaining were found in many adenocarcinomas arising elsewhere in the GI tract. They also demonstrated that primary and metastatic colorectal carcinomas showed remarkably similar scoring patterns. All primary and 25 of 26 metastatic colonic adenocarcinomas showed high levels of CDX2 expression (2+ or 3+) in this study. In another study, Kaimaktchiev et al [32] observed a greater than 80% concordance for CDX2 expression in the analysis of matched primary and lymph node metastases. In addition, all 17 colorectal metastases examined by whole sections were CDX2 positive in this study. Using tissue microarrays, Moskaluk et al [29] analyzed CDX2 staining in 745 samples of human cancer and arrived at similar conclusions. Barbareschi et al [30] compared CDX2 expression in primary and metastatic tumors found in the lung and concluded that this marker is highly selective for tumors originating from the colon and rectum, but also stains metastases from the stomach and ovary. In our study, CDX2 was expressed in 114 of 118 (97%) colorectal, 36 of 59 (61%) gastric, and 5 of 32(16%) pancreatic adenocarcinomas. The majority of cases (92/114, 81%) demonstrated strong and diffuse immunostaining in more than 50% of cells in colorectal tumors. Among the CDX2 positive gastric carcinomas (36/59), reactivity was focal in 22 cases (22/36, 61%). Among the 32 cases of pancreatic adenocarcinoma, only 5 cases were focally positive for CDX2.

Among colorectal adenocarcinomas, the relationship between tumor grade and CDX2 staining has been controversial. Hinoi et al [43] demonstrated that a rare subset of poorly differentiated colonic carcinomas termed large cell minimally differentiated carcinoma or medullary carcinoma are characterized by microsatellite instability and loss of CDX2 expression. Kaimaktchiev et al [32] recently studied tissue microarray samples of 1109 colorectal adenocarcinomas and found a lack of CDX2 reactivity in 14 (28%) of 50 poorly differentiated tumors. They concluded that CDX2 expression decreases with tumor differentiation. Other series, however, failed to find a strong correlation between CDX2 expression and the level of differentiation in colorectal adenocarcinomas. In the study of Werling et al [28], 74 of 75 colonic carcinomas showed high levels of CDX2 expression (2+ or 3+). Although several high-grade tumors showed scores of 2+ (26%-75% positive cells) compared with scores of 3+ (> 75% positive cells) that were observed in all well-differentiated carcinomas, the authors concluded that the expression of CDX2 did not appear to correlate with the level of tumor differentiation. Saad et al [31] also showed that CDX2 expression was not influenced by tumor grade. In this study, there was no significant association between CDX2 expression and tumor differentiation in colorectal carcinomas (98% of low grade tumors and 91% of high grade tumors were positive for CDX2). Our semiquantitative scoring system did not, however, take into account the intensity of immunostaining, but focused exclusively on the fraction of cells positively immunostained. It is likely that other methods of assessing absolute levels of CDX2 expression might show differences related to tumor differentiation.

CDX2 represents the latest in a series of transcription factors that have found important applications in diagnostic surgical pathology as highly specific and sensitive markers of specific cell and tumor types. Nuclear transcription factors have several distinct advantages over cytoplasmic ''differentiation'' markers: (1) transcription factors generally yield an ''all or none'' signal, with most of the positive cases containing positive signal in > 90% of the tumor cell population; (2) given the nuclear localization of the signal, it is much less likely to be confused with biotin or other sources of false positive cytoplasmic signals; and (3) lack of association between the levels of expression of nuclear transcription factors and the state of differentiation of the tumor [28]. For example, in the study described here, 114 of 118 cases of colonic adenocarcinoma were CDX2-positive, independent of tumor grade.

Expression of CDX2 in tumors other than colorectal carcinoma has been previously reported [28,29,32-35,40,41]. CDX2 expression has been documented in gastric adenocarcinoma by several different groups [28,32,43-46]. Werling et al [28] reported scores of 2+ (26%-75% positive cells) and 3+ (> 75% positive cells) positivity in 17 (70%) of 24 cases. These authors also reported that no association between any histological subtypes within pancreatic or gastric tumors and CDX2 expression could be discerned. In the study of Kaimaktchiev et al [32], CDX2 staining was observed in gastric adenocarcinomas (16 of 71), more commonly in the intestinal-type than in the diffuse-type (28.9 vs 11.5%). Our results are entirely consistent with these studies in that CDX2 staining was observed in 61% of gastric adenocarcinomas and significantly favored in the intestinal-type tumors over the diffuse variants (77% versus 45%). Park et al [44] reported that, CDX2 expression was decreased in early gastric cancers, when compared with dysplasia, and was even more reduced in advanced cancers. Similarly, Kim et al [45] reported lesser CDX2 expression in early gastric cancers compared to advanced tumors. Liu et al [46] also showed that CDX2 expression is progressively decreased in gastric intestinal metaplasia, dysplasia, and cancer. We didn't find any association between CDX2 expression and stage of gastric adenocarcinomas. As for CDX2 expression in pancreatic ductal adenocarcinomas, there appears to be somewhat less agreement in the literature. Werling et al [28] reported scores of 2+ (26%-75% positive cells) and 3+ (> 75% positive cells) positivity in 7 (32%) of 22 cases, Moskaluk et al [29] found 1+ ( < 25% positive cells) expression in 8 (33%) of 24 cases, and in the series of Chu et al [35], CDX2 reacted with 10 (22%) of 46 cases. In contrast, Kaimaktchiev et al [32] found that only 3 of 70 cases were positive for this marker. In the present study, among the 32 cases of pancreatic adenocarcinomas, only 5 cases were focally positive for CDX2.

Based on the studies mentioned above CDX2 expression alone does not reliably distinguish between colorectal adenocarcinomas and adenocarcinomas arising elsewhere in the GI tract, particularly pancreatobiliary and gastric adenocarcinomas, although the sensitivityof CDX2 for colorectal cancer is significantly higher than for these latter tumors. Qualitatively, focal and weak CDX2 expression in a given tumor favors extra-intestinal origin whereas uniform intense expression favors intestinal origin. In comparison with the CK7-/CK20+ immunoprofile Tot [47] found that CK7-/CK20+ expression pattern was more specific for colonic adenocarcinoma metastases than CDX2 alone (98.7% vs 90%), but less sensitive (79.5% vs. 84%). We also evaluated the sensitivity, specificity, positive predictive value, and negative predictive value of CDX2 expression and CK7-/CK20+ immunophenotype to differentiate colorectal adenocarcinoma from pancreatic and gastric adenocarcinomas. Determining the CK7/CK20 phenotype proved to be more specific in differentiating colorectal adenocarcinoma from pancreatic and gastric adenocarcinomas (specificity 96.7%) than the expression of CDX2 was. The CK7-/CK20+ phenotype had a superior positive predictive value (96.2%) in these circumstances. CDX2 expression at both cut-off levels (> 5% and > 50%) had a higher sensitivity (96.6% and 78%) and higher negative predictive value (92.6% and 74.8%) than the CK phenotype. The specificity of CDX2 expression did not reach the level of specificity of CK7/CK20 phenotype at a > 50% level, either (84.6%).

## Conclusions

Both the CK7-/CK20+ phenotype and expression of the antibody CDX2 are highly specific and sensitive markers of colorectal origin. CDX2 expression should be a useful adjunct for the diagnosis of intestinal adenocarcinomas, especially those with CK7+/CK20+ or CK7-/CK20- profiles. The CK7-/CK20+ immunophenotype is more specific in differentiating colorectal adenocarcinomas from pancreatic and gastric adenocarcinomas than CDX2 expression. The CK7-/CK20+ phenotype is superior in its specificity and positive predictive value and might be preferred.

## Competing interests

The authors declare that they have no competing interests.

## Authors' contributions

RB carried out the data collection, the pathological and immunohistochemical evaluation and interpretation, drafting and wrote the final manuscript; SY and HH participated in pathological and immunohistochemical evaluation and interpretation. All authors read and approved the final manuscript.
